# Cost‐effectiveness of a 12 country‐intervention to scale up short course TB preventive therapy among people living with HIV

**DOI:** 10.1002/jia2.25629

**Published:** 2020-10-26

**Authors:** Hyejeong Shin, Youngji Jo, Richard E Chaisson, Karin Turner, Gavin Churchyard, David W. Dowdy

**Affiliations:** ^1^ Department of Epidemiology Johns Hopkins Bloomberg School of Public Health Baltimore MD USA; ^2^ Center for Tuberculosis Research Johns Hopkins University School of Medicine Baltimore MD USA; ^3^ The Aurum Institute Parktown South Africa; ^4^ School of Public Health University of Witwatersrand Johannesburg South Africa

**Keywords:** TB/HIV, short‐course TB preventive therapy, three months of weekly rifapentine and isoniazid therapy, cost‐effectiveness analysis, catalytic impact, large‐scale initiative

## Abstract

**Introduction:**

In 2017, the Aurum Institute, with support from Unitaid, launched an initiative to expand short‐course therapy for the prevention of tuberculosis (TB) in 12 high‐burden countries. This study aimed to investigate the importance of “catalytic” effects beyond the original project timeframe when estimating cost‐effectiveness of such large investments.

**Methods:**

We estimated the cost‐effectiveness of the IMPAACT4TB (I4TB) initiative from a health system perspective, using a 10‐year time horizon. We first conservatively estimated costs using a “top‐down” approach considering only the direct health benefits of providing TB preventive therapy to people initiating antiretroviral therapy (ART) through I4TB activities. We then re‐estimated the incremental cost‐effectiveness of I4TB incorporating the costs and health benefits of potential catalytic effects beyond the program itself.

**Results:**

We estimated that TB preventive therapy through the I4TB initiative alone would prevent 14 201 cases of active TB and 1562 TB deaths over 10 years with an up‐front investment of $52.5 million; the estimated incremental cost‐effectiveness was $1580 per disability‐adjusted life year (DALY) averted. If this initiative could achieve its desired catalytic effects, an additional 375 648 cases and 41 321 deaths could be averted, at an incremental cost of $546 million and cost‐effectiveness of $713 per DALY averted.

**Conclusions:**

Our findings provide donors with reasonable evidence of value for money to support investment in short‐course TB preventive therapy for people initiating ART in high‐burden settings. Our study also illustrates the importance of considering long‐term secondary (“catalytic”) effects when evaluating the cost‐effectiveness of large‐scale initiatives designed to change a global policy landscape.

## 1 INTRODUCTION

Since 1993, the World Health Organization (WHO) has recommended preventive therapy for tuberculosis (TB) for people living with HIV (PLHIV) in whom active TB has been excluded (without testing for latent TB infection, LTBI) [1]. Yet the uptake of TB preventive therapy (TPT) – or treatment for LTBI – in high burden countries remains low [2]. In 2011, a novel short course regimen for TPT, consisting of three months of weekly rifapentine and isoniazid (3HP), was shown to have less toxicity, better adherence, lower risk of developing resistance and similar efficacy to six months of daily isoniazid (isoniazid preventive therapy, IPT) [3]. In 2018, WHO released updated guidelines recommending 3HP as an alternative to IPT for treating LTBI in high TB burden countries [4, 5].

In 2017, the Aurum Institute, with funding and support from Unitaid [6], launched the IMPAACT4TB (Increasing Market and Public health outcomes through scaling up Affordable Access models of short Course preventive Therapy for TB, I4TB) initiative to promote scale‐up of 3HP. This initiative allocated $59 million to provide 3HP to PLHIV and paediatric TB contacts across 12 high‐burden countries (Brazil, Cambodia, Ethiopia, Ghana, India, Indonesia, Kenya, Malawi, Mozambique, South Africa, Tanzania, Zimbabwe), representing 50% of global TB incidence. The I4TB project also includes comprehensive activities to support global scale‐up of 3HP, including negotiations to lower the price of rifapentine, supporting development of a generic fixed‐dose combination, coordination of procurement and support of global advocacy. Implementation of 3HP through I4TB is scheduled to start in late 2020 to early 2021.

When evaluating the value‐for‐money of large initiatives such as I4TB that aim not only to deliver an intervention (TPT) but also to change a broader global landscape, it is important to consider that a large up‐front investment may be necessary to facilitate future scale‐up activities at the country level. For example overcoming initial policy hurdles and demonstrating that short‐course TPT can successfully be implemented at scale may create a “catalytic” effect, in which TPT is delivered to a much greater population, both in the original I4TB countries and worldwide. Cost‐effectiveness analyses of such major initiatives should therefore explicitly consider the potential for such catalytic effects (and the uncertainty thereof). We conducted a cost‐effectiveness analysis of the I4TB project (with a focus on PLHIV) that considers the project both in isolation and incorporating its potential catalytic impact over a future 10‐year horizon.

## 2 METHODS

### 2.1 Estimating the direct effect of I4TB

We first sought to conservatively estimate the effectiveness and cost‐effectiveness of I4TB by only considering the impact of directly providing 3HP to 695 000 PLHIV newly enrolled on ART within the context of I4TB in the 12 target countries (the I4TB target population size). We estimated the number of TB cases and deaths that would be averted over a 10‐year time horizon, using probability parameters from the scientific literature (Table 1). We assumed that 23% of the target population would have LTBI (of whom 1.6% would reactivate TB each year), that 80% of the target population would complete 3HP (with 90% efficacy among those who complete), and that PLHIV on ART who subsequently develop active TB have 10% case‐fatality. For simplicity and conservativeness, we assumed that individuals receiving TPT were not recently infected with TB, and that TPT offered no benefit against TB disease due to reinfection after TPT initiation. We assumed that PLHIV initiating ART remain in care without loss to follow‐up for the entire 10‐year horizon. Future TB cases and deaths were converted to disability‐adjusted life years (DALYs) by assuming standard disability weights [7] and an average 27‐year life expectancy of HIV‐positive individuals on ART at the time of TPT. Future DALYs (and costs, below) were discounted at 3% per year, with sensitivity analysis between 0% and 7%. Calculations were performed in R version 3.5.3 (R Foundation for Statistical Computing, Vienna, Austria). All data and R scripts for reproducing this analysis are available at https://github.com/TB‐Modeling/impaact4tb‐tpt‐3hp.

**Table 1:** Parameter values

Epidemiologic values	Base	Low	High	Source
Prevalence of LTBI^a^	0.23	0.20	0.35	[26]
TB reactivation rate on ART^b^	0.016	0.012	0.020	[19, 20]
Efficacy of 3HP	0.9	0.8	0.95	[8]
Completion of 3HP	0.8	0.6	0.9	[8]
TB case‐fatality ratio for HIV + on ART^b^	0.1	0.08	0.14	[27]
Life expectancy of HIV + on ART^b^	27	20.25	33.75	[28]
TPT Toxicity without hospitalization^b^	0.082	0.062	0.103	[29]
TPT Toxicity with hospitalization^b^	0.003	0.002	0.004	[29]
Disability weights				
Disability weight, HIV on ART	0.053	0.034	0.079	[7]
Disability weight, TB/HIV	0.399	0.267	0.547	[7]
Costs^c^				
Cost per outpatient visit, Africa	$2.39	−50%	+50%	[11]
Cost per outpatient visit, Latin America/Asia	$2.65	−50%	+50%	[11]
Cost per hospital bed‐day, Africa	$12.84	−50%	+50%	[11]
Cost per hospital bed‐day, Latin America/Asia	$12.85	−50%	+50%	[11]
Laboratory testing for toxicity, Africa	$16.31	−50%	+50%	[10, 11]
Laboratory testing for toxicity, Latin America/Asia	$18.13	−50%	+50%	[10, 11]
TB treatment cost, Africa	$446	−50%	+50%	[13]
TB treatment cost, Latin America/Asia	$1040	−50%	+50%	[13]
3HP price after volume reaching 10 million doses	$10	$7	$12	Assumption
ART cost, per person‐year	$64	$61	$75	[12]
Discounting rate	0.03	0.00	0.07	Assumption

3HP, three months of weekly isoniazid and rifapentine therapy; ART, antiretroviral therapy; HIV, human immunodeficiency virus; LTBI, latent tuberculosis infection; TB, tuberculosis; TPT, TB preventive therapy.

^a^ We used a wider range of LTBI prevalence from 0.20 to 0.35 (reflecting the lower bound for the African region and the upper bound for the Southeast Asian region), given the large variation in LTBI prevalence across regions and countries.

^b^ For epidemiologic parameters where 95% uncertainty ranges were not available from the literature, we applied −25% of the base value as the lower bound and +25% of the base value as the upper bound.

^c^ For cost parameters, we applied −50% of the base value as the lower bound and +50% of the base value as the upper bound.

### 2.2 Cost and cost‐effectiveness of I4TB (Direct Effects Only)

We conservatively estimated the cost of providing TPT for PLHIV through I4TB as the “top‐down” budget of the entire initiative ($59 million), after subtracting the estimated portion ($6 million) for TPT among child contacts, based on the relative size of each target group. We excluded household contact investigation with TPT for paediatric contacts from this analysis because it represents a different intervention with different considerations for both costs and effectiveness. Our goal in this initial analysis was to be maximally conservative, not ignoring any costs that might be incurred. We therefore implicitly assumed that I4TB could not be performed without substantial overhead costs for project coordination, technical assistance, monitoring and evaluation and implementation research to inform models of delivery. In addition to the “top‐down” cost of I4TB, we also incorporated costs for adverse events, assuming that 8.2% of all PLHIV receiving TPT would experience a treatment‐limiting adverse event incurring costs of additional laboratory testing, and that 0.3% of individuals would experience an adverse event requiring seven days of hospitalization [8, 9]. We assumed a one‐time outpatient visit and laboratory testing (complete blood count, electrolyte panel, urinalysis and liver function tests) for all patients experiencing such toxicity, plus hospitalization costs for those requiring inpatient care. Country‐specific costs of laboratory testing were assumed to follow the same proportional cost, relative to South Africa (where laboratory costs were available [10]), as the cost of outpatient treatment. Costs of outpatient visits and hospitalization were estimated from WHO‐CHOICE [11]. We assumed that 3HP would save TB treatment costs for those in whom TB reactivation was averted; we also included additional ART costs (estimated at $64 per year [12]) for those in whom TB death was averted. We also considered, in the case of dolutegravir, the potential need for additional dosing with rifapentine; this did not alter our estimates of cost‐effectiveness by more than 1% (data not shown). TB treatment cost in each country was estimated based on its gross domestic product (GDP) per capita, using WHO’s TB treatment cost regression model (TB treatment cost = e^−2.2 + 1.1*ln(GDP)^) [13]. This formula accounts for the higher cost of TB treatment in wealthier countries where labour is more expensive. In all cost analyses, we took an average of each country‐specific cost by region, converted costs into same‐year US dollars and inflated those costs to 2018 using the US GDP deflator [14]. When costs or savings occurred in the future, we calculated the net present value of all future costs and savings accruing to each patient in the year in which s/he was given 3HP.

In our primary analysis, we compared 3HP to no TPT based on historical evidence of low IPT uptake in these countries. (In other words, we assumed that TPT delivered through I4TB would be additional to existing levels of TPT rather than replacing IPT or other TPT regimens.) Our primary outcome was the incremental cost‐effectiveness ratio (ICER), comparing the intervention to the existing status quo, assuming that TPT coverage under the status quo would remain constant at 2019 levels in the absence of I4TB. Data were collected from the scientific literature and the project implementing agency from 29 July 2019 to 11 February 2020.

### 2.3 Estimating the catalytic effect of I4TB

Following this maximally conservative cost‐effectiveness estimate of the I4TB initiative above, we next estimated the potential “catalytic” impact of I4TB between 2020 and 2030. We assumed that I4TB would enable countries to enhance provision of TPT to 32 million PLHIV, both those newly enrolling and already enrolled in care globally by 2030. We calculated this total simulated population size by taking the current global prevalence of HIV and assuming that the mean annual decline in new HIV infections over the preceding 10 years [15] would continue linearly for the next decade. We further assumed a two‐year delay between new HIV infection and diagnosis, and constant 81% ART coverage (90% of all PLHIV know their status, of whom 90% are on treatment) in future years.

To project annual 3HP coverage by 2030 with potential catalytic impact not only within 12 target countries but also worldwide, we categorized countries into three groups based on their anticipated potential to open a window for policy change according to the framework of Kingdon [16]. This framework conceptualizes the potential for policy change as a function of the size of the perceived problem, availability of effective policy and favourability of the political environment. We characterized problem size based on TB incidence and TB/HIV comorbidity, TPT policy availability based on existing coverage of TPT and ART, and favourability of the political environment based on the per‐capita budget for TB/HIV as a proportion of per‐capita gross national income (GNI). Using these data, we classified countries into those with “high,” “moderate,” and “low” potential for TPT policy change following I4TB (Appendix S1). We then assumed that countries with “high” potential would reach 90% TPT coverage by 2025 and 95% by 2030 for both PLHIV on ART and PLHIV newly enrolled in care; countries with “moderate” potential would reach 90% TPT coverage by 2030; and countries with “low” potential would reach 70% TPT coverage by 2030. We assumed linear increases in TPT coverage over the post‐I4TB time period. Specific TPT coverage assumptions by the group are provided in Appendix S2.

### 2.4 Cost and cost‐effectiveness of I4TB, incorporating catalytic effects

We assumed that, once a 3HP delivery system has been set up in each country through the mechanism of I4TB, 3HP could be delivered to patients for the cost of drugs plus three outpatient visits and a 25% markup for overheads including procurement and delivery, monitoring and evaluation and ongoing quality assurance. We used the recently discounted price of 3HP ($15 per adult course), reflecting negotiations by Sanofi and Unitaid/Global Fund [17] followed by manufacture of a generic fixed‐dose combination. We assumed that this price would fall to $12 in 2022 and subsequently stabilize until reaching a global volume of 10 million doses (in 2024 under our projection), at which time we assumed the price could be reduced to $10 per course.

Using the estimates of cost and effectiveness as described above, we re‐estimated the incremental cost‐effectiveness of I4TB considering both the direct impact of the project itself (using maximally conservative estimates, as above) and the potential catalytic impact. We again adopted a 10‐year horizon for all costs and effects, assuming that policymakers might use 10 years as a reasonable horizon for decision making. To provide budgetary estimates, we also estimated costs required and saved plus cases and deaths averted in each year without discounting.

### 2.5 Sensitivity analysis

We performed one‐way sensitivity analyses to describe the association between each input variable and the primary outcome (i.e. incremental cost‐effectiveness of I4TB). To explore the simultaneous effect of uncertainty in all model parameters, we also conducted a probabilistic sensitivity analysis (PSA) in which all model parameter values were randomly sampled over pre‐specified distributions (shown in Table 1). This process was repeated 10 000 times to generate uncertainty estimates around the primary cost‐effectiveness estimate, with 95% uncertainty ranges (95% UR) reported as the 2.5^th^ and 97.5^th^ percentiles of the corresponding distributions.

To the extent that I4TB‐delivered 3HP would replace IPT (rather than add to existing TPT efforts), the incremental catalytic impact of I4TB would be reduced. As such, we performed a secondary analysis in which 50% of PLHIV receiving 3HP would otherwise have received IPT. To explore the impact and cost‐effectiveness of I4TB over a longer time horizon, we additionally considered a 27‐year time horizon (as the mean life expectancy of patients receiving 3HP). We also performed a sensitivity analysis with each country group (Appendix S3) and with a higher LTBI prevalence (31%) which was seen in the Southeast Asian region [18] (Appendix S4).

### 2.6 Ethical statement

Neither ethical approval nor informed consent was required for this analysis which did not involve human subjects research.

## 3 RESULTS

Under our initial conservative analysis, we estimated that, if I4TB could effectively deliver 3HP to 695 707 PLHIV initiating ART, it would avert 14 201 (95% UR: 8097 to 19 367) cases of active TB, 1562 (799 to 2326) deaths, and 29 368 (12 405 to 52 311) DALYs over a 10‐year time horizon compared to the status quo. After discounting and removing the budget for child contacts, the top‐down cost of I4TB was $52.5 million. We estimated that $8.1 million would be saved due to averted treatment costs, but an additional $2 million would be required for management of toxicity and incremental ART for people avoiding TB death, resulting in an incremental cost of $46.4 million and incremental cost‐effectiveness of $1580 ($859 to $3940) per DALY averted over a 10‐year horizon (Table 2). This cost‐effectiveness estimate fell by 44% (to $890 per DALY averted) when using a 27‐year horizon.

**Table 2:** Cost‐effectiveness of the I4TB initiative over a 10‐year time horizon

Scenario	Cost (2018 US dollars, in millions)					Effectiveness and Cost‐effectiveness			
	TB Preventive Therapy	Averted TB Treatment	Additional ART	3HP Toxicity	Incremental cost	TB cases averted	TB deaths averted	DALYs averted	ICER (cost per DALY averted)
Direct effect (n = 695 707)	$52.5	−$8.1	$0.5	$1.5	$46.4	14 201	1562	29 368	$1580
Incremental catalytic effect^a^ (n = 31 918 629)	$676.6	−$208.6	$17.1	$60.9	$545.9	375 648	41 321	800 889	–
Catalytic impact	$729.0	−$216.7	$17.7	$62.4	$592.3	389 849	42 883	830 257	$713

3HP, three months of weekly isoniazid and rifapentine therapy; ART, antiretroviral therapy; DALY, disability adjusted life years; I4TB, IMPAACT4TB initiative; ICER, incremental cost‐effectiveness ratio; TB, tuberculosis.

^a^ We assumed that countries with “high” potential would reach 90% TPT coverage by 2025 and 95% by 2030 for both currently and newly enrolled PLHIV on ART; countries with “moderate” potential would reach 90% TPT coverage by 2030; and countries with “low” potential would reach 70% TPT coverage by 2030. We applied a linear increase in TPT coverage in the period from 2020 to 2030.

### 3.1 Cost‐effectiveness considering catalytic impact

If the I4TB initiative could catalyse TPT delivery to an additional 32 million PLHIV over 10 years, an additional 389 849 (219 488 to 540 370) cases of active TB, 42 883 (21 522 to 64 523) deaths, and 830 257 (340 901 to 1 502 158) DALYs would be averted relative to the status quo over that time frame. The additional cost of providing 32 million courses of 3HP was estimated at $677 million, resulting in $209 million in averted treatment costs and $78 million in additional costs for toxicity management and ART in survivors. As a result, after also considering this catalytic impact, the estimated incremental cost‐effectiveness of I4TB fell to $713 ($370 to $1781) per DALY averted (Table 2). If the baseline catalytic effect was cut in half (e.g. if 50% PLHIV receiving 3HP would otherwise have received IPT or if the number of PLHIV receiving TPT only met 50% of initial projections), the estimated incremental cost‐effectiveness of I4TB rose to $743 per DALY averted.

**Figure 1:**
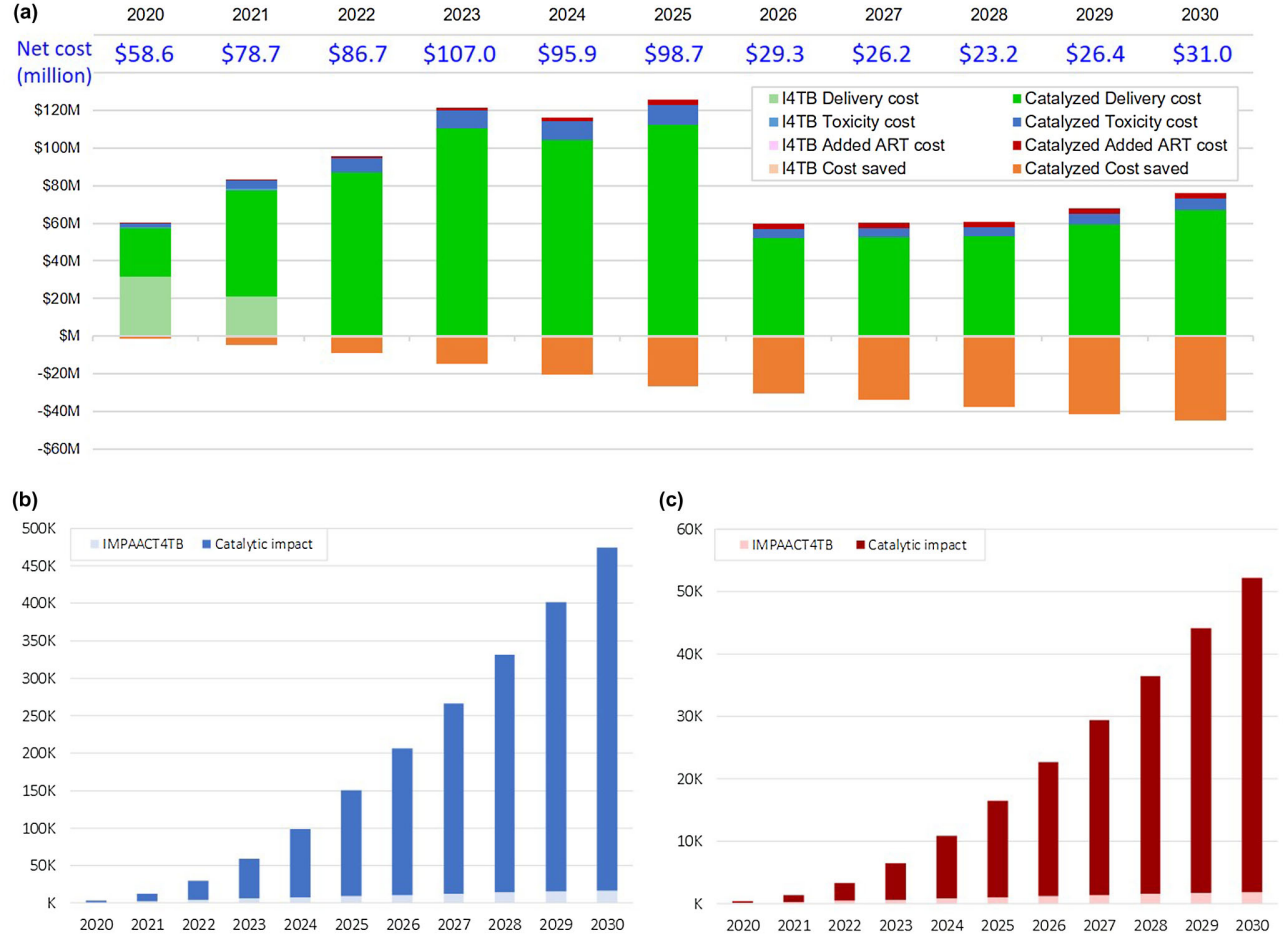
Budget impact of I4TB, 2020 to 2030. **(a)** Shows costs and benefits (costs saved) from the direct and catalyzed impact of I4TB by calendar year (i.e. without discounting). Direct costs of I4TB (light green) are high in the initial two years, reflecting the large initial investment in the I4TB initiative itself. After 2026, the catalyzed delivery costs (dark green) were substantially lower on a per‐person basis. Toxicity treatment costs (light and dark blue) for adverse events and additional ART costs required for people in whom TB death is averted (light and dark red) are also considered in this budgetary analysis. TB treatment costs saved (light and dark orange) reflect TB cases that are averted by TPT. **(b)** Shows the cumulative number of TB cases averted by 2030 considering the direct effect of I4TB (light blue) and the additional catalytic impact (dark blue). **(c)** Shows similar numbers but for TB deaths averted.

Figure 1 presents the estimated budget impact (as undiscounted costs and benefits) of I4TB through 2030, broken down according to direct and catalysed effects. The I4TB‐catalysed delivery of 3HP would require an additional budgetary outlay of $780 million over 10 years, beyond the initial $53 million investment by Unitaid. However, when considering TB treatment costs saved, annual net costs for “catalysed” TPT after 2026 would be less than half of the first‐year investment. Moreover, additional cost savings would be realized in years beyond 2030 (Figure 1).

### 3.2 Sensitivity analysis

**Figure 2:**
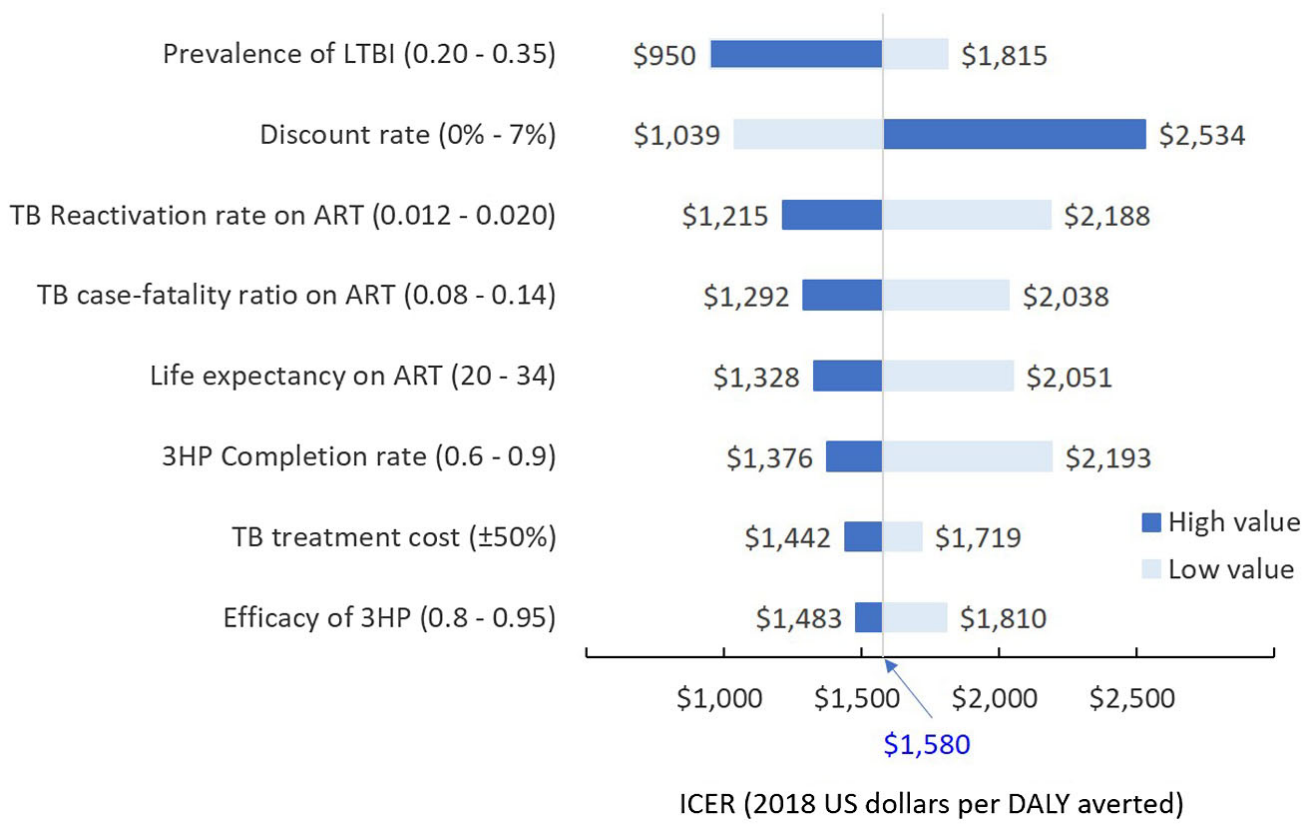
One‐way sensitivity analysis of I4TB (Direct effect). The parameters shown had the greatest absolute influence (among parameters evaluated in the model) on the incremental cost‐effectiveness ratio (ICER) of the I4TB intervention in one‐way sensitivity analyses. Bars show the ICER (incremental dollars per DALY averted in 2018 US dollars) of the I4TB intervention under variation of each parameter over the range specified, with the dark blue bar representing the high parameter value and light blue bar representing the low parameter value, holding the values of all other parameters as constant. For example, we varied the TB reactivation rate by 0.012 to 0.02 from the baseline (0.016), which caused the ICER to vary from its baseline value of $1580/DALY averted to $2188/DALY averted (assuming a lower reactivation rate) and $1215/DALY averted (assuming a higher reactivation rate). Cost‐effectiveness estimates shown here are for the direct impact of I4TB only (no catalytic costs), based on top‐down cost estimates (Unitaid total budget allocation) with a 10‐year time horizon. Variables for which the ICER did not vary by more than ±$100 were excluded from the figure.

**Figure 3:**
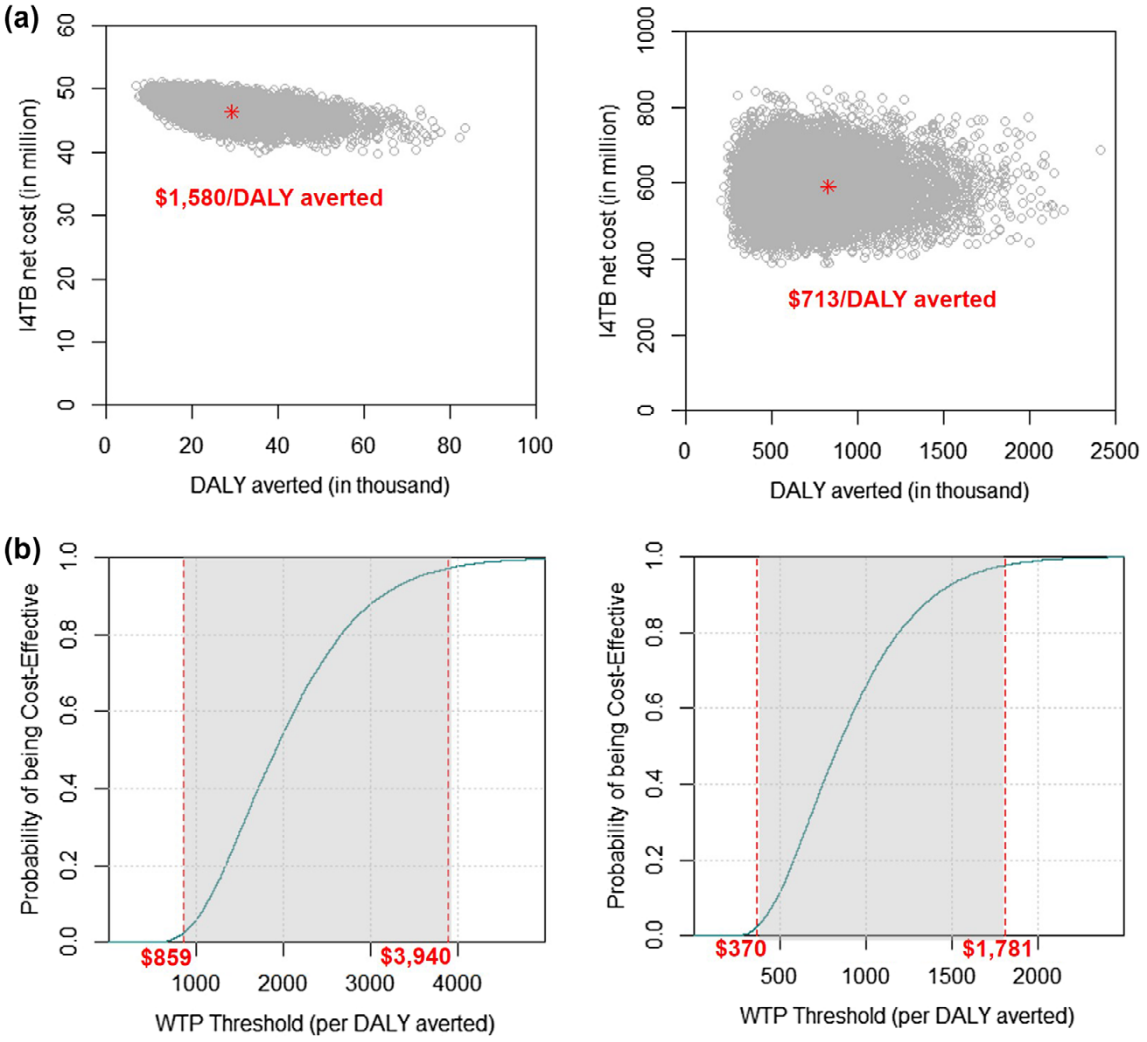
Cost‐effectiveness of I4TB: Probabilistic sensitivity analysis. The cost effectiveness planes **(a)** depict the simulated outputs from probabilistic sensitivity analyses for direct effect (left) and catalytic effect (right) of I4TB. The horizontal axis denotes the disability adjusted life years (DALYs) averted in each simulation, and the vertical axis indicates the incremental costs of the I4TB initiative compared to the status quo. Costs are expressed in 2018 US dollars. The cost effectiveness planes indicate the uncertainty around the incremental cost effectiveness ratio, in terms of both costs (variation on the y‐axis) and DALYs averted (variation on the x‐axis). In the cost effectiveness acceptability curves **(b)**, the horizontal axis denotes the willingness to pay (WTP) per DALY averted (incremental cost‐effectiveness ratio, ICER), and the vertical axis indicates the probability of cost‐effectiveness based on the proportion of simulations in which the comparison of the I4TB intervention to the baseline falls below the WTP threshold shown on the x‐axis. The gray area in each curve indicates the 95% uncertainty range, or the range between the 2.5^th^ and 97.5^th^ percentiles of simulations, in terms of their calculated cost per DALY averted. Costs are again expressed in 2018 US dollars.

Figure 2 illustrates the model parameters that were most influential on the primary outcome of I4TB cost‐effectiveness, in the direct‐effect‐only scenario. Specifically, variation in the assumed discount rate, TB reactivation rate and probability of 3HP completion alone could cause the estimated cost‐effectiveness of I4TB to rise as high as $2200‐$2500 per DALY averted. The results of probabilistic sensitivity analysis are shown in Figure 3, illustrating that uncertainty regarding the effectiveness of I4TB is greater than uncertainty in cost. Despite this uncertainty, the probability that I4TB would be considered cost‐effective at a threshold of $4000 per DALY averted, even in the most conservative scenario of top‐down costing and a 10‐year horizon of effects, was 98%. After incorporating potential catalytic effects, the probability of cost‐effectiveness at a threshold of $1000 per DALY averted was 67%, and at a threshold of $2000 per DALY averted was 99%.

If the cost of rifapentine is dramatically reduced to an unlikely price of $1 per course upon achieving a global volume of 10 million doses, the estimated incremental cost‐effectiveness becomes $474 per DALY averted under the catalytic impact scenario. When we estimate the cost‐effectiveness with a 27‐year time horizon, I4TB with catalytic impact prevents at least 402 841 TB cases and 44 313 TB deaths, resulting in incremental cost‐effectiveness as low as $690 per DALY averted.

## 4 DISCUSSION

This analysis of I4TB illustrates the importance of considering long‐term secondary (“catalytic”) effects when evaluating the cost‐effectiveness of interventions that require an initial up‐front investment to change an existing policy and practice landscape. For example we estimated that, after discounting, the I4TB initiative alone could prevent about 14 000 cases of active TB and 1600 TB deaths over 10 years with an up‐front investment of $52.5 million, at a maximally conservative cost‐effectiveness estimate of $1580 per DALY averted. If this effort could catalyse TPT provision to an additional 32 million PLHIV, 376 000 cases of active TB and 41 000 deaths due to TB could be averted additionally in the same 10 years, at an incremental cost of $546 million and cost‐effectiveness of $713 per DALY averted. This incremental cost corresponds to less than 1% of currently allocated global budgets for TB and HIV [2, 18]. Cost‐effectiveness was enhanced even further under a longer time horizon or lower price of rifapentine. These analyses suggest that I4TB is likely to be cost‐effective at most widely used willingness‐to‐pay thresholds and more broadly illustrate the importance of incorporating catalytic effects when estimating the cost‐effectiveness of major global health initiatives.

In estimating the cost‐effectiveness of any large initiative with desired secondary effects, there will be some uncertainty about how much of the planned catalytic impact will actually be achieved. It is therefore important to consider cost‐effectiveness across different scenarios of this potential catalytic impact, as we have done. For example using very simple calculations, we estimate an incremental cost‐effectiveness of $1580 per DALY averted in the absence of any catalytic impact, $743 per DALY averted if half the planned catalytic impact is achieved, and $713 per DALY averted if the full anticipated catalytic impact is achieved. These estimates can be used by funders to evaluate the anticipated cost‐effectiveness of their investment based on their desired cost‐effectiveness threshold and willingness to assume risk regarding catalysed effects. This approach of providing transparent and straightforward estimates of cost‐effectiveness under different scenarios of secondary impact can also serve as a useful template for funding bodies who are considering making similar large investments in global health in the future.

The I4TB initiative includes 12 countries representing very different economic and epidemiological contexts. The cost‐effectiveness of I4TB is sensitive to assumptions about TB epidemiology and the cost of treatment that are likely to differ across countries [8, 9, 19, 20, 21]; individual countries are also likely to apply different cost‐effectiveness thresholds in their decision making. To generate a single estimate of cost‐effectiveness for this multi‐country intervention, we were forced to combine data across all countries into a single summary estimate; however, decisions of whether to scale up 3HP will be made at a country level, and country‐specific estimates of budget impact and cost‐effectiveness are also important to provide.

Previous studies have emphasized the importance of estimating costs considering economies of scale when evaluating cost‐effectiveness of large interventions. For example Gauvreau *et al*. [22] argued that most studies of paediatric immunization programs should extend their time horizons for evaluating the impacts of donor funding because implementation costs of such programs tend to be incurred predominantly at the beginning of intervention scale‐up. As further examples, Menzies *et al*. [23] and Gupta *et al*. [24] showed that unit costs of ART delivery fall by 50% or more in the years following initial scale‐up, and Suwanthawornkul *et al*. [25] demonstrated that HPV vaccination, while costing $1400 per DALY in the baseline scenario, could become cost‐saving when assuming universal coverage and applying economies of scale. These results illustrate the importance of considering economies of scale but do not explicitly evaluate large initiatives (such as I4TB) that are undertaken with the express intent of catalysing broad‐scale uptake of an underutilized intervention.

As with any model‐based analysis, this research has limitations. To provide a maximally conservative estimate of cost‐effectiveness that incorporates all possible costs of I4TB, we used a top‐down costing approach. However, the total cost of the I4TB initiative includes a wide range of indirect activities such as a safety and pharmacokinetic study for co‐administering 3HP with ART and supporting civil society engagement. Our primary results may therefore provide pessimistic estimates of the cost‐effectiveness of I4TB. Second, for purposes of transparency and of illustrating an approach that could be undertaken in time to inform decision making, we adopted a number of simplifying assumptions regarding the natural history of HIV and TB, future trajectory of the HIV epidemic, ART coverage and adherence and costs of delivering TPT and treatment for active TB. While this approach may be useful for high‐level decision making, our results should not be misinterpreted as precise estimates of the cost‐effectiveness of I4TB or of 3HP. To provide more accurate estimates, incorporation of more detailed country‐specific data would be necessary. Third, we used a very conservative 10‐year time horizon, reasoning that decision makers might not be strongly influenced by effects or budget impact occurring beyond this time frame; use of a longer (e.g. lifetime) time horizon would greatly enhance the estimated cost‐effectiveness of the intervention. Finally, we applied simple and optimistic assumptions regarding future 3HP coverage as a result of catalytic impact, which might bias our cost‐effectiveness estimates if those volumes are not achieved. However, even if only half of the desired catalytic impact is achieved, overall cost‐effectiveness estimates remained similar ($743 vs $713 per DALY averted).

## 5 CONCLUSION

In conclusion, this study provides quantitative estimates of the cost‐effectiveness of a multi‐country initiative aimed at changing the policy landscape regarding preventive therapy for TB among people living with HIV. Our results underscore the importance of considering “catalytic” impact when estimating the cost‐effectiveness of large‐scale interventions that require substantial up‐front investment to facilitate future implementation, which in turn generates health benefits and cost savings over a longer period of time.

## COMPETING INTEREST

All authors have no potential conflicts of interest.

## AUTHORS’ CONTRIBUTIONS

HS, YJ and DD designed the research study and developed the model. KT and GC contributed the data. HS and YJ performed analysis with supervision from DD. HS wrote the first draft of the manuscript. YJ, RC, KT, GC and DD provided interpretation and provided important intellectual input. HS, YJ, RC, GC and DD reviewed and revised the manuscript. All authors have read and approved the final manuscript.

## Supporting information


**Table S1.** Country classification
**Table S2.** Annual 3HP coverage projection by I4TB and catalytic impact
**Table S3.** Group‐specific cost‐effectiveness of the I4TB initiative over a 10‐year time horizon
**Table S4.** Cost‐effectiveness of the I4TB initiative with higher prevalence of LTBI (31%)* over a 10‐year time horizon

## References

[1] World Health Organization . Tuberculosis preventive therapy in HIV‐infected individuals. A Joint Statement of the WHO Tuberculosis Programme and the Global Programme on AIDS, and the International Union Against Tuberculosis and Lung Disease (IUATLD). Wkly Epidemiol Rec. 1993;68(49):361–4. 8292529

[2] World Health Organization . Global tuberculosis report 2018. Geneva: World Health Organization; 2018.

[3] Martinson NA , Barnes GL , Moulton LH , Msandiwa R , Hausler H , Ram M , et al. New regimens to prevent tuberculosis in adults with HIV infection. N Engl J Med. 2011;365(1):11–20. 21732833 10.1056/NEJMoa1005136 PMC3407678

[4] World Health Organization . Latent TB Infection : Updated and consolidated guidelines for programmatic management. Geneva: World Health Organization; 2018. 30277688

[5] World Health Organization . Meeting of the Implementation Core Group of WHO Global Task Force on Latent TB Infection and country stakeholders on implementation tools and joint TB and HIV programming to scale up TB preventive treatment. Geneva: World Health Organization; 2018[cited 2019 Nov 1]. Available from: https://www.who.int/tb/publications/2018/latent‐tuberculosis‐infection/en/

[6] IMPAACT4TB . IMPAACT4TB [Internet]. 2018 [cited 2020 Jan 10]. Available from: https://www.impaact4tb.org/

[7] Global Burden of Disease Collaborative Network . Global burden of disease study 2010 (GBD 2010) disability weights. Seattle: Institute for Health Metrics and Evaluation (IHME); 2012.

[8] Sterling TR , Elsa VM , Borisov AS , Shang N , Gordin F , Bliven‐Sizemore E , et al. Three months of rifapentine and isoniazid for latent tuberculosis infection. New Engl J Med Med. 2011;365(23):2155–66. 10.1056/NEJMoa1104875 22150035

[9] Belknap R , Holland D , Feng P‐J , Millet J‐P , Caylà JA , Martinson NA , et al. Self‐administered versus directly observed once‐weekly isoniazid and rifapentine treatment of latent tuberculosis infection: a randomized trial. Ann Intern Med. 2017;167(10):689–97. 29114781 10.7326/M17-1150 PMC5766341

[10] Naidoo K , Grobler AC , Deghaye N , Reddy T , Gengiah S , Gray A , et al. Cost‐effectiveness of initiating antiretroviral therapy at different points in TB treatment in HIV‐TB co‐infected ambulatory patients in South Africa. J Acquir Immune Defic Syndr. 2015;69(5):576–84. 26167618 10.1097/QAI.0000000000000673 PMC4503368

[11] World Health Organization . WHO‐CHOICE unit cost estimates for service delivery [Internet]. 2008 [cited 2019 Nov 10]. Available from: http://www.who.int/choice/country/WHO‐CHOICEunit_cost_estimates_2007_2008.xls

[12] MSF Access Campaign . Stopping Senseless Deaths. Technical Briefing Document. 2018.

[13] World Health Organization . Global Tuberculosis Report 2016. Geneva: World Health Organization; 2016.

[14] World Bank . World Bank National Accounts data, and OECD National Accounts data files: GDP deflator (Malawi) [Internet]. [cited 2019 Jul 29]. Available from: https://data.worldbank.org/indicator/NY.GDP.DEFL.ZS?locations=MW

[15] World Bank . HIV estimates with uncertainty bounds 1990‐2018 [Internet]. [cited 2019 Oct 1]. Available from: https://www.unaids.org/en/resources/documents/2019/HIV_estimates_with_uncertainty_bounds_1990‐present

[16] Kingdon JW . How do issues get on public policy agendas? In: Wilson WJ, editor. Sociology and the public agenda. Washington DC: SAGE Publications, Inc; 1993 pp. 40–50.

[17] COWLHA, CRAG, DACASA, Global TB CAB, GAPA Rs .Jointed Hands Welfare Organization, et al. A Lower Price for Rifapentine Is Just a Start — Communities Need More Than Discounts to Access TB Preventive Therapy ! In Hyderabad, India: Treatment Action Group. 2019[cited 2019 Nov 12]. Available from: http://www.treatmentactiongroup.org/content/lower‐price‐rifapentine‐just‐start‐communities‐need‐more‐than‐discounts

[18] Houben RMGJ , Dodd PJ . The Global Burden of Latent Tuberculosis Infection: A Re‐estimation Using Mathematical Modelling. PLoS Med [Internet]. 2016;13(10):1–13. 10.1371/journal.pmed.1002152 PMC5079585 27780211

[19] UN Joint Programme on HIV/AIDS (UNAIDS) . UNAIDS DATA 2019. Geneva: 2019.

[20] Golub JE , Saraceni V , Cavalcante SC , Pacheco AG , Moulton LH , King BS , et al. impact of ART and IPT on TB in PLHIV Brazil. AIDS. 2007;21(11):1441–8. 17589190 10.1097/QAD.0b013e328216f441 PMC3063947

[21] Sterling TR , Scott NA , Miro JM , Calvet G , La Rosa A , Infante R , et al. Three months of weekly rifapentine and isoniazid for treatment of Mycobacterium tuberculosis infection in HIV‐coinfected persons. AIDS. 2016;30(10):1607–15. 27243774 10.1097/QAD.0000000000001098 PMC4899978

[22] Odone A , Amadasi S , White RG , Cohen T , Grant AD , Houben RMGJ . The impact of antiretroviral therapy on mortality in hiv positive people during tuberculosis treatment: a systematic review and meta‐analysis. PLoS One. 2014;9(11):1–12. 10.1371/journal.pone.0112017 PMC4229142 25391135

[23] Gauvreau C , Ungar WJ , Hler JICK , Zlotkin S . The use of cost‐effectiveness analysis for pediatric immunization in developing countries. Milbank Q. 2012;90(4):762–90. 23216430 10.1111/j.1468-0009.2012.00682.x PMC3530741

[24] Menzies NA , Berruti AA , Berzon R , Filler S , Ferris R , Ellerbrock TV , et al. The cost of providing comprehensive HIV Treatment in PEPFAR‐supported programs. AIDS. 2011;25(14):1753–60. 21412127 10.1097/QAD.0b013e3283463eec PMC3225224

[25] Gupta I , Trivedi M , Kandamuthan S . Recurrent costs of India’s free ART program In: Haacker M, Claeson M, editors. HIV and AIDS in South Asia: an economic development risk. Washington: World Bank; 2009 pp. 191–237.

[26] Suwanthawornkul T , Praditsitthikorn N , Kulpeng W , Haasis MA , Guerrero AM , Teerawattananon Y . Incorporating economies of scale in the cost estimation in economic evaluation of PCV and HPV vaccination programmes in the Philippines : a game changer? Cost Eff Resour Alloc [Internet]. 2018;1–16. 10.1186/s12962-018-0087-x 29483848 PMC5819712

[27] Odone A , Amadasi S , White RG , Cohen T , Grant AD , Houben RMGJ . The impact of antiretroviral therapy on mortality in HIV positive people during tuberculosis treatment. Syst Rev Meta Anal. 2014;9:11. 10.1371/journal.pone.0112017 PMC4229142 25391135

[28] Mills EJ , Bakanda C , Birungi J , Chan K , Ford N , Cooper CL , et al. Life expectancy of persons receiving combination antiretroviral therapy in low‐income countries: a cohort analysis from Uganda. Ann Intern Med. 2011;155(4):209–17. 21768555 10.7326/0003-4819-155-4-201108160-00358

[29] Shepardson D , Marks SM , Chesson H , Kerrigan A , Holland DP , Scott N , et al. tuberculous infection in the United States. Int J Tuberc Lung Dis. 2013;17(12):1531–7. 24200264 10.5588/ijtld.13.0423 PMC5451112

