# Supplementary material for: Cost‐effectiveness of a 12 country‐intervention to scale up short course TB preventive therapy among people living with HIV
Source: J Int AIDS Soc. 2020 Oct 26;23(10):e25629. doi: 10.1002/jia2.25629 (PMC7588607; doi:10.1002/jia2.25629)
Supplement: Supplementary file 1 — Table S1. Country classification Table S2. Annual 3HP coverage projection by I4TB and catalytic impact Table S3. Group‐specific cost‐effectiveness of the I4TB initiative over a 10‐year time horizon Table S4. Cost‐effectiveness of the I4TB initiative with higher prevalence of LTBI (31%)* over a 10‐year time horizon [file JIA2-23-e25629-s001.docx]

**Supporting information**

**S1. Country classification**

| **Group** | **Potential for TPT policy change** | **Problem** | | **Policy** | | **Politics** | |
| --- | --- | --- | --- | --- | --- | --- | --- |
|  |  | **TB Incidence**^a^ | **TB/HIV comorbidity**^a^ | **IPT coverage**^a^ | **ART coverage**^b^ | **budget (TB + HIV) per capita**^c^ | **I4TB effect**^d^ |
|  |  | rate (per 100K) | % | % | % | % of GNI per capita | % |
| African countries within I4TB | High | 237 | 27% | 32% | 60% | 73% | Direct |
| African countries out-of-I4TB | High | 237 | 27% | 32% | 60% | 73% | Direct |
|  | Moderate | 237 | 27% | 32% | 60% | 73% | Indirect |
| Asian countries within I4TB | Moderate | 226 | 3% | 12% | 46% | 51% | Direct |
| Latin American countries within I4TB | Moderate | 28 | 11% | 16% | 60% | 85% | Direct |
| Asian countries out-of-I4TB | Low | 226 | 3% | 12% | 46% | 51% | Limited |
| Latin American countries out-of-I4TB | Low | 28 | 11% | 16% | 60% | 85% | Limited |

^a^ Each value indicates regional value from Global TB report 2018 (1).

^b^ Each value indicates regional value from UNAIDS DATA 2019 (2).

^c^ Each value indicates regional value from Global TB report 2018 (TB budget) (1) and UNAIDS DATA 2019 (HIV budget) (2).

^d^ Political environment of 12 countries covered by I4TB is directly exposed to a novel short course regimen for TPT (3HP); we assumed that 50% of African countries outside of I4TB coverage would be also directly exposed to 3HP since they are geographically adjacent to I4TB countries. The rest of 50% African countries outside of I4TB was assumed to be indirectly effected on 3HP political environment by I4TB. The rest of countries were assumed to be limited impact on 3HP political environment by I4TB.

**S2. Annual 3HP coverage projection by I4TB and catalytic impact**

| **Potential for TPT policy change** | **High** | **Moderate** | **Low** |
| --- | --- | --- | --- |
| **Target** | **90% by 2025, 95% by 2030** | **90% by 2030** | **70% by 2030** |
| **Currently enrolled PLHIV in care** | | | |
| 2020 | 5% | 0% | 0% |
| 2021 | 15% | 5% | 0% |
| 2022 | 30% | 10% | 2% |
| 2023 | 50% | 15% | 5% |
| 2024 | 70% | 20% | 10% |
| 2025 | 90% | 30% | 15% |
| 2026 | 91% | 40% | 25% |
| 2027 | 92% | 50% | 35% |
| 2028 | 93% | 60% | 45% |
| 2029 | 94% | 75% | 55% |
| 2030 | 95% | 90% | 70% |
| **Newly enrolled PLHIV in care** | | | |
| 2020 | 50% | 50% | 0% |
| 2021 | 60% | 50% | 0% |
| 2022 | 60% | 60% | 30% |
| 2023 | 70% | 64% | 35% |
| 2024 | 80% | 68% | 40% |
| 2025 | 90% | 71% | 45% |
| 2026 | 91% | 75% | 50% |
| 2027 | 92% | 79% | 55% |
| 2028 | 93% | 83% | 60% |
| 2029 | 94% | 87% | 65% |
| 2030 | 95% | 90% | 70% |

**S3. Group-specific cost-effectiveness of the I4TB initiative over a 10-year time horizon**

| Country | Cost (2018 US dollars, in thousands) | | | | | Effectiveness and Cost-effectiveness | | | |
| --- | --- | --- | --- | --- | --- | --- | --- | --- | --- |
|  | TB Preventive Therapy | Averted  TB Treatment | ART cost added | Toxicity cost | Incremental cost | Cases averted | Deaths averted | DALYs averted | ICER  (Cost per DALY averted) |
| Direct effect | | | | | | | | | |
| African countries  within I4TB  (n=548,069) | 41.3M | 5.0M | 0.4M | 1.2M | 37.9M | 11,185 | 1,230 | 23,130 | 1,641 |
| Non-African countries  within I4TB  (n=147,637) | 11.1M | 3.1M | 0.1M | 0.3M | 8.5M | 3,017 | 332 | 6,239 | 1,356 |
| Incremental Catalytic effect | | | | | | | | | |
| African countries  within I4TB  (n=14,806,306) | 326M | 93M | 9M | 28M | 271M | 207,624 | 22,839 | 440,084 | - |
| Non-African countries  within I4TB  (n=2,721,065) | 56M | 25M | 1M | 5M | 37M | 24,450 | 2,689 | 52,713 | - |
| African countries  out-of-I4TB  (n=8,595,912) | 180M | 44M | 5M | 16M | 156M | 98,761 | 10,864 | 210,633 | - |
| Non-African countries  out-of-I4TB  (n=5,795,346) | 115M | 47M | 2M | 11M | 82M | 44,812 | 4,929 | 97,459 | - |
| Catalytic Impact | | | | | | | | | |
| African countries  within I4TB  (n=15,354,375) | 379M | 101M | 10M | 30M | 318M | 221,826 | 24,401 | 469,452 | 677 |
| Non-African countries  within I4TB  (n=2,868,702) | 108M | 34M | 2M | 7M | 83M | 38,651 | 4,252 | 82,082 | 1,016 |
| African countries  out-of-I4TB  (n=8,595,912) | 232M | 52M | 5M | 17M | 202M | 112,963 | 12,426 | 240,001 | 843 |
| Non-African countries  out-of-I4TB  (n=5,795,346) | 167M | 55M | 3M | 13M | 128M | 59,014 | 6,492 | 126,827 | 1,011 |
| **Total** | **729M** | **217M** | **18M** | **62M** | **592M** | **389,849** | **42,883** | **830,257** | **713** |

**S4. Cost-effectiveness of the I4TB initiative with higher prevalence of LTBI (31%)* over a 10-year time horizon**

| Scenario | Cost (2018 US dollars, in millions) | | | | | Effectiveness and Cost-effectiveness | | | |
| --- | --- | --- | --- | --- | --- | --- | --- | --- | --- |
|  | TB Preventive Therapy | Averted  TB Treatment | Addi-  tional  ART | 3HP Toxicity | Incre-mental  cost | TB  cases  averted | TB deaths averted | DALYs averted | ICER  (Cost per  DALY averted) |
| Direct effect  (n=695,707) | $52.5 | -$10.9 | $0.7 | $1.5 | $43.8 | 19,141 | 2,106 | 39,583 | $1,106 |
| Incremental  Catalytic effect ^a^  (n=31,918,629) | $676.6 | -$281.2 | $23.1 | $60.9 | $479.3 | 506,308 | 55,694 | 1,079,459 |  |
| Catalytic impact | $729.0 | -$292.1 | $23.8 | $62.4 | $523.1 | 525,449 | 57,799 | 1,119,042 | $467 |

*Using higher prevalence of LTBI in Southeast Asia Region (3), we re-calculated cost-effectiveness of the I4TB initiative over a 10-year time horizon.

**Reference**

1. World Health Organization. Global Tuberculosis Report 2018. Geneva; 2018.

2. UN Joint Programme on HIV/AIDS (UNAIDS). UNAIDS DATA 2019. Geneva; 2019.

3. Houben RMGJ, Dodd PJ. The Global Burden of Latent Tuberculosis Infection: A Re-estimation Using Mathematical Modelling. PLoS Med [Internet]. 2016;13(10):1–13. Available from: http://dx.doi.org/10.1371/journal.pmed.1002152
